# Straightening Beta: Overdispersion of Lethal Chromosome Aberrations following Radiotherapeutic Doses Leads to Terminal Linearity in the Alpha–Beta Model

**DOI:** 10.3389/fonc.2017.00318

**Published:** 2017-12-21

**Authors:** Igor Shuryak, Bradford D. Loucas, Michael N. Cornforth

**Affiliations:** ^1^Center for Radiological Research, Columbia University, New York, United States; ^2^Department of Radiation Oncology, University of Texas Medical Branch, Galveston, TX, United States

**Keywords:** linear-quadratic model, stereotactic radiotherapy, radiation, chromosomal aberrations, lethal lesions, survival curve, overdispersion

## Abstract

Recent technological advances allow precise radiation delivery to tumor targets. As opposed to more conventional radiotherapy—where multiple small fractions are given—in some cases, the preferred course of treatment may involve only a few (or even one) large dose(s) per fraction. Under these conditions, the choice of appropriate radiobiological model complicates the tasks of predicting radiotherapy outcomes and designing new treatment regimens. The most commonly used model for this purpose is the venerable linear-quadratic (LQ) formalism as it applies to cell survival. However, predictions based on the LQ model are frequently at odds with data following very high acute doses. In particular, although the LQ predicts a continuously bending dose–response relationship for the logarithm of cell survival, empirical evidence over the high-dose region suggests that the survival response is instead log-linear with dose. Here, we show that the distribution of lethal chromosomal lesions among individual human cells (lymphocytes and fibroblasts) exposed to gamma rays and X rays is somewhat overdispersed, compared with the Poisson distribution. Further, we show that such overdispersion affects the predicted dose response for cell survival (the fraction of cells with zero lethal lesions). This causes the dose response to approximate log-linear behavior at high doses, even when the mean number of lethal lesions per cell is well fitted by the continuously curving LQ model. Accounting for overdispersion of lethal lesions provides a novel, mechanistically based explanation for the observed shapes of cell survival dose responses that, in principle, may offer a tractable and clinically useful approach for modeling the effects of high doses per fraction.

## Introduction

At traditional doses per fraction (e.g., <8 Gy) used for cancer radiotherapy, the linear-quadratic (LQ) model of cell killing by radiation continues to be successfully used. This simple formalism has a mechanistic justification in terms of lethal lesions produced by single ionizing tracks (intratrack action) versus those produced by multiple independent tracks (intertrack action). A notable feature of the LQ model, as it is usually applied, is that it presupposes that the number of lethal lesions per cell is Poisson distributed. For low LET radiations, the LQ dose response for the mean number of lethal lesions per cell exhibits a characteristic upward curvature throughout the entire dose range. With a few possible exceptions (1–3), the LQ model stands in contrast to the large (and diverse) majority of biophysical models of radiation action developed over the years, which predict linearity in the mean frequency of lesions per cell at high doses, and consequently, a “terminal exponential tail” in the dose response for the logarithm of cell survival (4–7).

In fact, cell survival data often produce dose responses that approach a constant slope on a logarithmic scale (“terminal exponential”) (8, 9) As such, they are in seeming conflict with the predictions of the LQ model. Several approaches have been used to mitigate this apparent discrepancy, some involving modifications of the LQ formalism with various degrees of mechanistic motivation (10), whereas others appear largely or essentially *ad hoc* by design (11).

A common feature of these approaches is that they focus on the *mean* number of lethal lesions per cell as function of radiation dose, where the error distribution around the mean is assumed to be Poisson. In other words, different models have different formulations for the dose dependence of the mean lethal lesion yield, but, to our knowledge, all of them rely on the Poisson distribution to calculate the surviving fraction (i.e., the fraction of cells with zero lethal lesions). Such assumptions are understandable, given that these models evolved to explain clonogenic cell survival, an endpoint for which the *number* of lesions in individual cells cannot be quantified.

For the current studies, we take advantage of the well-established relationship between cytogenetic damage and cell killing (12). For example, under experimentally controlled conditions, there is a virtual 1:1 relationship between cells harboring certain aberration types (i.e., dicentrics, rings, interstitial deletions, and terminal deletions) and cell lethality measured by colony-forming assays (13). Of additional importance, cytogenetic analysis allows damage to be measured with great precision on a cell-by-cell basis, meaning that the distribution of lethal lesions can be quantified among irradiated cells.

Although the Poisson distribution is a reasonable approximation for lethal lesion data, there are reasons to believe that it may not be the best choice for therapeutically relevant doses of sparsely ionizing radiation (e.g., gamma rays and X rays with energies typically used in radiation oncology). For example, the microdosimetric distribution of radiation energy deposition is not optimally approximated by the Poisson distribution (14). The stochastic nature of the number of ionizing track traversals per cell and the number of DNA double-strand breaks (DSBs) induced by each of these tracks can also lead to non-Poisson behavior, which are often modeled by compound Poisson (Neyman) distributions (15, 16). Chemical and biological factors, such as heterogeneity of DSB complexities, the existence of multiple DSB repair pathways with different fidelities (17, 18), and diversity of lethal aberration classes, can also contribute to deviations from Poisson expectations. It is plausible to hypothesize that these processes can lead to substantial deviations from the 1:1 variance to mean ratio assumed by the Poisson distribution. In fact, situations where the variance is larger than the mean—so-called overdispersion—are common in count data from various fields (15, 19–21). For example, we have shown (22) that even a single track from gamma rays is capable of producing complex aberrations involving up to four chromosomes; this, by definition, will lead to overdispersion (15).

Overdispersion of radiation-induced lethal lesion yields is not merely of theoretical interest, but can be clinically important for cancer radiotherapy. This is because overdispersion alters the relationship between the mean number of lethal lesions per cell and survival (the probability of a cell having zero lethal lesions). It follows that even if the *mean* lethal lesion yield is described by the same function of dose, changing the *error distribution* from Poisson to an overdispersed alternative can change the predicted cell survival curve shape.

Here, we used data on clonogenically lethal chromosomal aberrations in human cells (lymphocytes and fibroblasts) exposed to gamma rays and X rays to search for overdispersion and to quantify clinically relevant effects that overdispersion may have on survival curve shape. Our objectives are to show the following: (1) There is indeed cytogenetic evidence for overdispersion of lethal lesions per cell. (2) The estimated overdispersion is sufficient to produce effectively log-linear behavior of the cell surviving fraction at high doses, even if the mean number of lethal lesions per cell is generated from the continuously curving LQ model. We also argue that accounting for overdispersion may therefore have clinically relevant implications for explaining and predicting radiotherapy effects at high doses per fraction.

## Materials and Methods

### Cytogenetic Data

We quantified the numbers of clonogenically lethal chromosomal lesions in human lymphocytes exposed to 4 Gy of gamma rays (23) as follows. A dicentric or centric ring (together with any associated compound acentric fragment) was considered a single lethal lesion. Each acentric fragment not associated with an exchange (i.e., terminal deletion) was considered a lethal lesion, as was each interstitial deletion. We also used previously published data on lethal chromosomal lesions in human fibroblasts exposed to 0–12 Gy of X rays (13). This data set contained only the mean number of lethal lesions per cell and the fraction of cells with zero lethal lesions at each dose, whereas the full distribution of lesions per cell was not reported. Cell culture, irradiations, and cytogenetic procedures have been previously described in detail (13, 24).

For the lymphocytes data, blood was drawn from two healthy male volunteers following procedures approved by the UTMB Institutional Review Board. Written informed consent was obtained from the participants of this study. Cells were cultured in 25-cm^2^ tissue culture flasks containing 5 ml RPMI 1640 medium supplemented with 15% fetal bovine serum plus penicillin and streptomycin. To this was added 0.1 ml phytohemagglutinin and 0.4 ml whole blood. Immediately thereafter, cells were irradiated using a JL Shepherd Mark 69-1 Irradiator with 4 Gy of ^137^Cs gamma rays at a dose rate of 1.3 Gy/min. Irradiation cells were incubated for 48 h before harvest with 0.1 µg/ml Colcemid, present during the final 3 h. Cells were then fixed and spread onto slides following standard cytogenetic procedures. Slides were processed for mFISH hybridization using SpectraVision 24-color probe cocktail (Vysis). Images were captured using a Zeiss Axiophot epifluorescence microscope equipped with a black and white CCD camera controlled by Power Gene image analysis software (Applied Imaging, Inc.). Karyotypes were constructed from these images, and exchanges were scored. These were assigned mPAINT descriptors as described previously (25). In cases where ambiguity existed in the classification of complex exchanges, these were brought to completion in the most conservative way possible to achieve multicolor “pattern closure.” The number of breakpoints associated with these exchanges were recorded and used for subsequent analyses. Cells devoid of acentric fragments (to include terminal deletions and fragments associated with complex and simple exchanges) were considered survivable.

For the fibroblast data, low-passage AG1522 normal human fibroblasts were obtained from the NIA cell repository. To prepare samples for metaphase chromosome analysis, two 6-h Colcemid (0.1 µg/ml) collection intervals were used that encompassed the time range spanning the peak of the first-division mitotic index. The first collection interval was between 30 and 36 h after subculture and the last between 36 and 42 h. Following the addition of 0.075 M KC1, cell suspensions were fixed onto glass microscope slides using standard cytogenetic methods to include staining in Sorensen’s-buffered Giemsa. Barring the infrequent occurrence of tricentric chromosomes, Giemsa staining does not directly allow for the identification of complex exchange aberrations. It is, nevertheless, capable of indirectly detecting many types of complex exchanges as pseudosimple exchanges (26). Cells without asymmetrical exchanges or fragments (including terminal deletions) were considered destined to survive.

### Models

We modeled the mean yield *Y* of clonogenically lethal chromosomal lesions per cell after an acute radiation dose *D* using the LQ formalism as follows:

(1) $$Y=\text{α}\times D+\text{β}\times D^{2}.$$

Here, α represents the contribution of lesions resulting from the same ionizing track, and β represents the contribution from different tracks. For simplicity, we ignore the small probability of lethal lesions in cells under background conditions (at *D* = 0).

The Poisson distribution is often used to model the distribution of lethal lesions per cell. It predicts the probability *P*_Pois_(*k*) of observing *k* lethal lesions in a cell as follows:

(2) $$P_{\text{Pois}}(k)=Y^{k}\times \text{exp}\left[-Y\right]/k!.$$

A fundamental property of the Poisson distribution is that its variance is equal to the mean. The negative binomial (NB) distribution is frequently used as a more flexible alternative that allows the variance to be larger than the mean. Here, we employed a customized NB distribution parametrization described in the Appendix (Eq. A1). The variance is described by the convenient expression *Y* + *rY*^2^. Consequently, if *r* approaches 0, there is no overdispersion and the variance and mean are equal, as in the Poisson distribution. On the other hand, if *r* > 0, the variance becomes greater than the mean and the ratio of variance to mean increases as the mean increases.

This specific formulation was chosen merely as a convenient example of an overdispersed distribution that is sufficiently general to represent a variety of mechanisms for overdispersion (e.g., the effects of microdosimetric energy deposition heterogeneity, DSB complexity, and repair pathway differences). Other distributions targeted to more specific mechanisms have also been used: e.g., the compound Poisson (Neyman) distribution, which accounts for stochasticity of the number of ionizing track traversals per cell and the number of chromosomal aberrations per track (15, 16).

The clonogenic cell surviving fraction *S* can be defined as the probability of zero lethal lesions. It can be calculated from each distribution as follows: *S*_NB_ = *P*_NB_(0) and *S*_Pois_ = *P*_Pois_(0). The solutions are as follows:

(3) $$S_{\text{NB}}={\left(1+r\times Y\right)}^{\left(-\frac{1}{r}\right)},S_{\text{Pois}}=\text{exp}\left[-Y\right].$$

We used maximum likelihood estimation to fit the Poisson and NB distributions to the data on gamma-ray irradiated lymphocytes. This popular approach involves finding parameter values that maximize the likelihood of making the observations given the parameters. For the Poisson distribution, the only adjustable parameter is the mean, *Y*. Its best-fit value for a particular data set can be found by maximizing the sum of log-likelihood (LL_Pois_) values across all analyzed cells. The log-likelihood function is described in the Appendix (Eq. A2). The same approach was applied to the NB distribution, where the mean (*Y*) and overdispersion (*r*) are adjustable parameters. The NB log-likelihood function is described in the Appendix (Eq. A3). Log-likelihood maximization was performed using sequential quadratic programming implemented in Maple 2016^®^ software.

To fit the data on fibroblasts, for which the full distribution of lethal lesions per cell was not available (13), we used the Binomial distribution to approximate the log-likelihood for the fraction of cells with zero lethal lesions. This was done as described in the Appendix (Eq. A4).

Ninety-five percent confidence intervals (CIs) for the adjustable parameters in each analyzed distribution were estimated by profile likelihood.

### Information Theoretic Comparison of Model Performances

Relative performances of different probability distributions fitted to the same data were assessed by the Akaike information criterion with sample size correction (AICc) (27). The equations for AICc and other information theoretic metrics are provided in the Appendix (Eqs A5–A7).

### Estimation of Biologically Effective Doses (BEDs)

Biologically effective dose is a convenient and frequently used metric for comparing the predicted potency of radiotherapy protocols with different dose fractionation schemes. Assuming complete radiation damage repair between dose fractions, we can calculate the BED for the *M*th-modeled distribution (BED*_M_*) as follows, where *N*_frac_ is the number of dose fractions and *S_M_* is calculated using Eq. 3 and substituting *Y* from Eq. 1:

(4) $${\text{BED}}_{M}=-N_{\text{frac}}\times \text{ln}\left[S_{M}\right]/\text{α}.$$

Explicit BED solutions for the Poisson and NB distributions are below, where *D* is dose per fraction:

(5) $$\begin{array}{ll} {\text{BED}}_{\text{NB}} & =\frac{N_{\text{frac}}}{\text{α}\times r}\times \text{ln}\left[1+r\times D\times (\text{α}+\text{β}\times D)\right],\\ {\text{BED}}_{\text{Pois}} & =N_{\text{frac}}\times D\times \left(1+\frac{\text{β}}{\text{α}}\times D\right). \end{array}$$

## Results

Summary statistics suggested that the distribution of lethal chromosomal lesions per cell in human lymphocytes exposed to 4 Gy of gamma rays was somewhat “overdispersed”: the mean was 1.96 and the variance was 2.51, so the ratio of variance/mean was ~1.3, rather than 1.0 as in the Poisson distribution. The upper 95% CI for skewness based on 10,000 bootstrap samples from the observed distribution was 2.16-fold greater than expected for the Poisson distribution.

**Figure 1:**
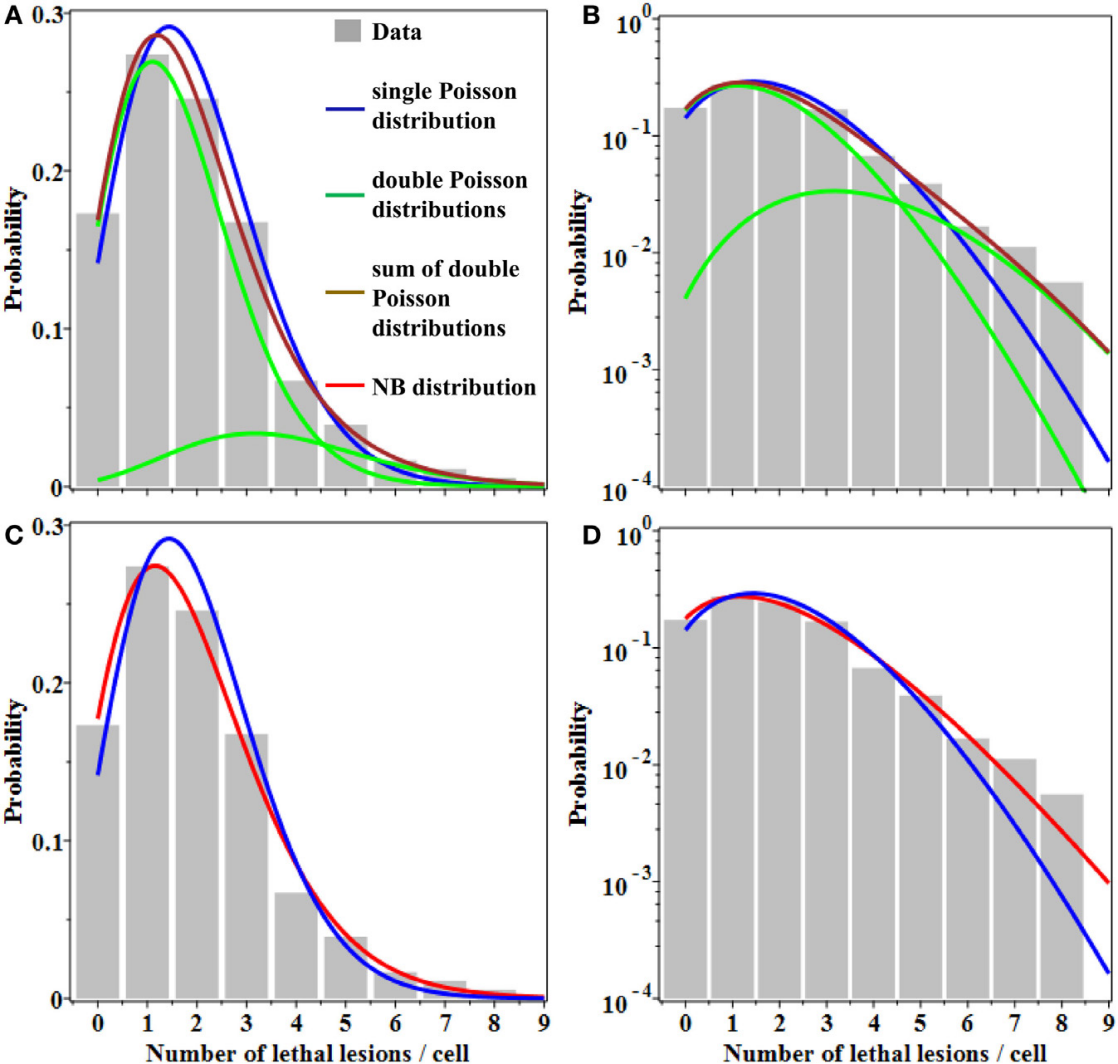
Best fits of different probability distributions to data on lethal lesions in lymphocytes exposed to 4 Gy of gamma rays. Gray bars, data; blue curve, single Poisson distribution; green curves, double Poisson distributions; brown curve, sum of double Poisson distributions; red curve, NB distribution. **(A,C)** A linear *y*-axis scale and **(B,D)** a logarithmic *y*-axis scale. The data are the same in all panels. Curves with the same color represent the same model fits in all panels.

Evidence for overdispersion was also found when we compared fits to the data from: (a) single Poisson distribution, (b) double Poisson distributions, and (c) the NB distribution. As shown graphically in Figure 1, a single Poisson distribution (with a best fit mean of 1.96) underestimated the “upper tail” of the observed distribution: the frequencies of 6–8 lesions per cell were considerably higher than the Poisson distribution would predict. Double Poisson distributions (with a first mean of 1.62 and a weight of 83.5%, and a second mean of 3.64 and a weight of 16.5%) outperformed single Poisson distribution by 1.8 AICc units. The NB distribution (with a mean of 1.96 and an *r* of 0.138) outperformed single Poisson distribution by 3.4 AICc units. The 95% CIs for the NB overdispersion parameter *r* did not overlap 0; they were 0.020 to 0.301.

Therefore, the NB and double Poisson distributions produced better fits than the single Poisson, particularly for the “upper tail” region of the data (Figure 1). The Akaike weights for all three models were 0.11 for single Poisson distribution, 0.27 for double Poisson distributions, and 0.62 for the NB distribution. The combined Akaike weight of overdispersed distributions was, therefore, 0.27 + 0.62 = 0.89.

Evidence for overdispersion of lethal lesions was also found at relevant doses in another cell type: human fibroblasts (13). After 6, 9, and 12 Gy of X rays, the mean numbers of lethal lesions per cell were 1.99, 3.51, and 5.42, and the corresponding observed fractions of cells with zero lethal lesions at these doses were 16.0, 4.0, and 1.3%, respectively (13). The Poisson distribution using observed mean values at each dose underestimated these fractions of cells with zero lethal lesions: it predicted 13.7, 3.0, and 0.4%, respectively. In contrast, the NB distribution with a best-fit *r* parameter of 0.078 (which was obtained by fitting all available data from 0 to 12 Gy) was considerably closer to these data at 6–12 Gy: it predicted 15.7, 4.5, and 1.1% zero lethal lesion probabilities. The Akaike weight was 0.59 for the NB distribution and correspondingly 0.41 for the Poisson distribution. The 95% CIs for NB parameter *r* were quite wide in this analysis, from 0.00 to 0.189. Notably, they overlapped the 95% CIs of 0.020 to 0.301 from lymphocyte data.

These results from lymphocytes and fibroblasts provide support for our contention that the distribution of lethal chromosomal lesions per cell at high doses of sparsely ionizing radiation (e.g., gamma rays) may not be optimally described by the Poisson distribution. Alternative approaches such as the NB distribution, which allow the variance to be larger than the mean, allow better fits to such data.

**Figure 2:**
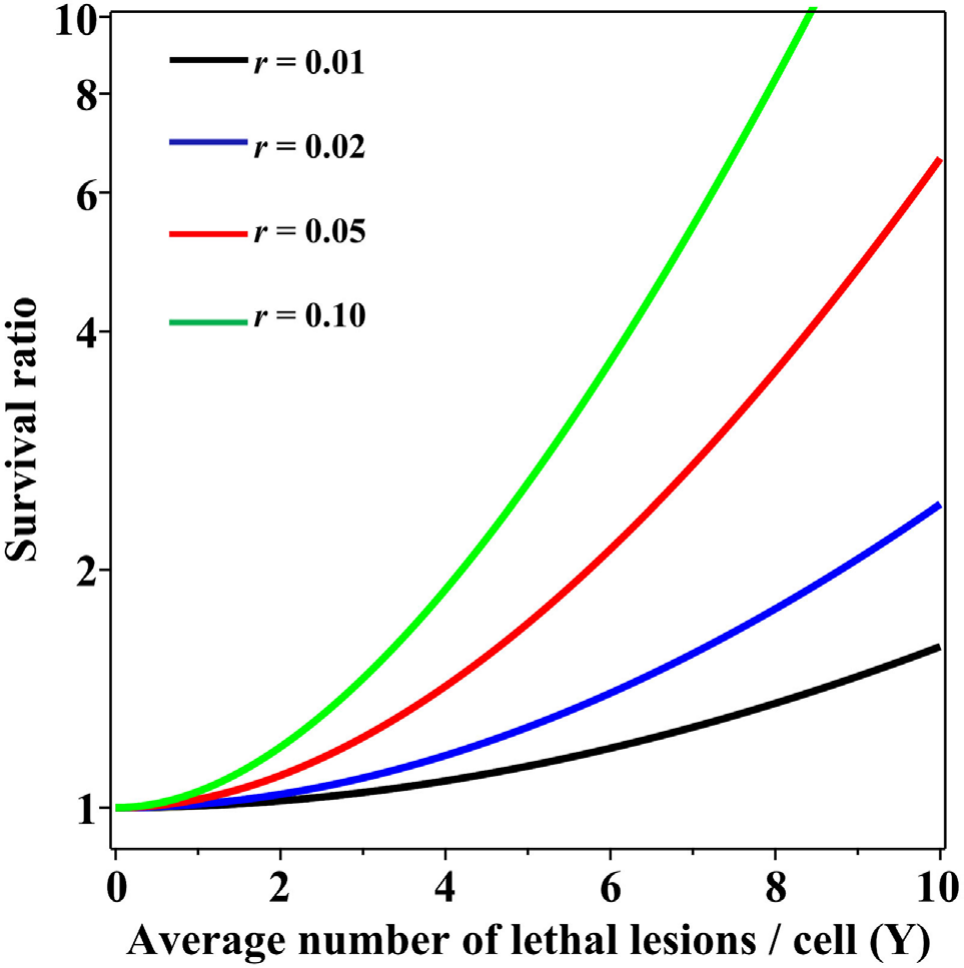
Ratio of cell surviving fractions (probabilities to have zero lethal lesions) predicted by the negative binomial (NB) distribution, relative to those predicted by the Poisson distribution, as function of the average number of lethal lesions per cell. This ratio is defined as *S*_NB_/*S*_Pois_ from Eq. 3 in the main text. Black curve, NB overdispersion parameter *r* = 0.01; blue curve, *r* = 0.02; red curve, *r* = 0.05; green curve, *r* = 0.10.

Importantly, when the Poisson and NB distributions have the same mean number of lethal lesions per cell, the probability of a cell to contain zero lethal lesions predicted by the NB distribution is larger than that predicted by the Poisson distribution. This effect increases rapidly with increasing mean number of lethal lesions per cell and with increasing overdispersion (NB parameter *r*; Figure 2). So, even when the overdispersion parameter *r* is constant, the ratio of variance to mean increases with increasing mean: the overdispersion, compared with the Poisson distribution, is therefore dose dependent. The important consequence is that at radiation doses that kill most cells by producing a large mean number of lethal lesions per cell, the Poisson distribution can predict substantially lower cell survival probabilities than the overdispersed NB distribution. A similar effect on the cell surviving fraction would also be produced by other overdispersed distributions, e.g., the compound Poisson (e.g., Neyman type A).

## Discussion

**Figure 3:**
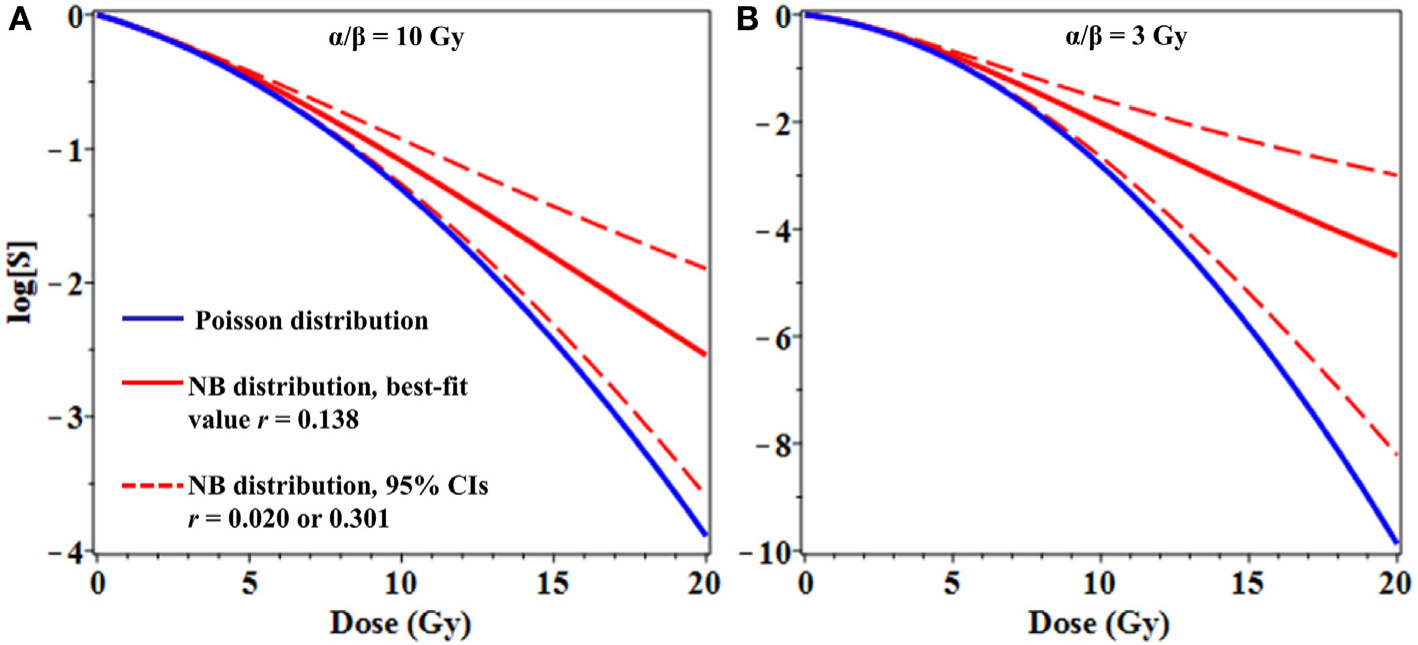
Effects of overdispersion of lethal lesions on cell survival curve shapes. The mean number of lethal lesions per cell in all curves was calculated using the linear-quadratic model with α = 0.15 Gy^−1^ and α/β = 10 Gy **(A)** or 3 Gy **(B)**. Blue curves, Poisson distribution of lethal lesions per cell; red curves, NB distribution. Solid red curves, negative binomial (NB) overdispersion parameter *r* = 0.138, the best-fit value for data on lethal lesions in lymphocytes exposed to 4 Gy of gamma rays. Dashed red curves, *r* set to the lower and upper 95% confidence intervals (CIs; 0.020 and 0.301, respectively) from the fit to these lymphocyte data.

From a theoretical perspective, binary misrepair serves as the cornerstone concept of the LQ formalism and the theory of dual radiation action (TDRA), upon which its mathematical underpinnings rest (28). Setting the TDRA model apart from most other models of radiation action is the notion that lesion pairs (formally sublesion pairs) combine to produce lethal cellular lesions. As is applies to cytogenetic theory, these represent the initial radiogenic breaks in chromosomes and the exchange-type aberrations that result. The intratrack and intertrack sources of these lesion pairs give rise to the linear (αD) and quadratic (βD^2^) components of the dose–response relationship, respectively. As mentioned, the use of mean values for lethal lesions in the LQ model tends to produce fits that bend continuously downward and away from actual survival data at high doses. As shown, consideration of the distribution of lesions among cells mitigates this tendency, producing fits that approach a terminal log-linear survival response (Figure 3) even if the mean lesion yield per cell is continuously curving with dose (LQ). In our minds, this serves to deflect a substantial criticism of binary misrepair as a fundamental concept in radiobiology.

The LQ model can be contrasted to more sophisticated and complex radiobiological models that emphasize biological repair kinetics, as opposed to spatial/temporal aspects of damage with respect to track structure. We take the view that the intratrack and intertrack concepts embodied in the LQ model serve to “set the stage” for subsequent repair/misrepair processes following initial radiogenic damage. For the purposes of predicting responses of cells or tissues exposed to acute radiation doses, it is debatable whether a detailed consideration of such repair processes is necessary.

There is no question that the concept of binary misrepair evolved from early radiation cytogenetic theory (29, 30), particularly as it relates to the formation of exchange-type aberrations, which make up the majority of lethal types. Unfortunately, complications arise when we take the term “binary” literally. This is because at very high doses, much (if not most) chromosome damage (e.g., exchange breakpoints) will not derive from simple pairwise exchanges between two chromosomes. Instead, they are likely to derive from complex exchanges, involving multiple chromosomes and breakpoints. Complex exchanges frequently give rise to multiple lethal events in a given cell. Simple n-th order polynomials cannot be used to model the dose response for exchange aberrations, because of competition for breakpoints that occurs during their genesis. For example, complex exchanges involving four radiogenic breaks compete with exchanges involving three breaks, which also compete for the breaks during the formation of simple two-break exchanges (31). As a result, from the perspective of modern cytogenetics, the β (dose-squared) term of the LQ model becomes little more than an ill-defined gross interactive term devoid of literal meaning.

From a clinical perspective, even modest differences in distribution shapes for lethal lesions per cell can have a potentially important effect on cell survival curve shapes at high radiation doses used for stereotactic radiotherapy (Figure 3). As a simple example, consider the case where the mean number of lethal lesions per cell is described by the LQ model with an α/β ratio of 10 Gy, which is not atypical many tumor types (Figure 3A), or 3 Gy, which is reasonable for late normal tissue complications (Figure 3B) (32, 33). When Poisson-distributed errors are used with this LQ model, a strongly downwardly curving survival curve is produced (blue curves in Figure 3). In contrast, using the same LQ model with NB-distributed errors—where the overdispersion parameter *r* was taken from the best-fit to gamma-irradiated lymphocyte data shown in Figure 1—resulted in substantially different survival curve shape at doses ≥8 Gy (Figure 3). Specifically, using NB-distributed errors “straightened” the survival curve at high doses. Such “straightening” is more consistent with *in vitro* cell survival data (8) than the behavior of the LQ model with Poisson-distributed errors.

**Figure 4:**
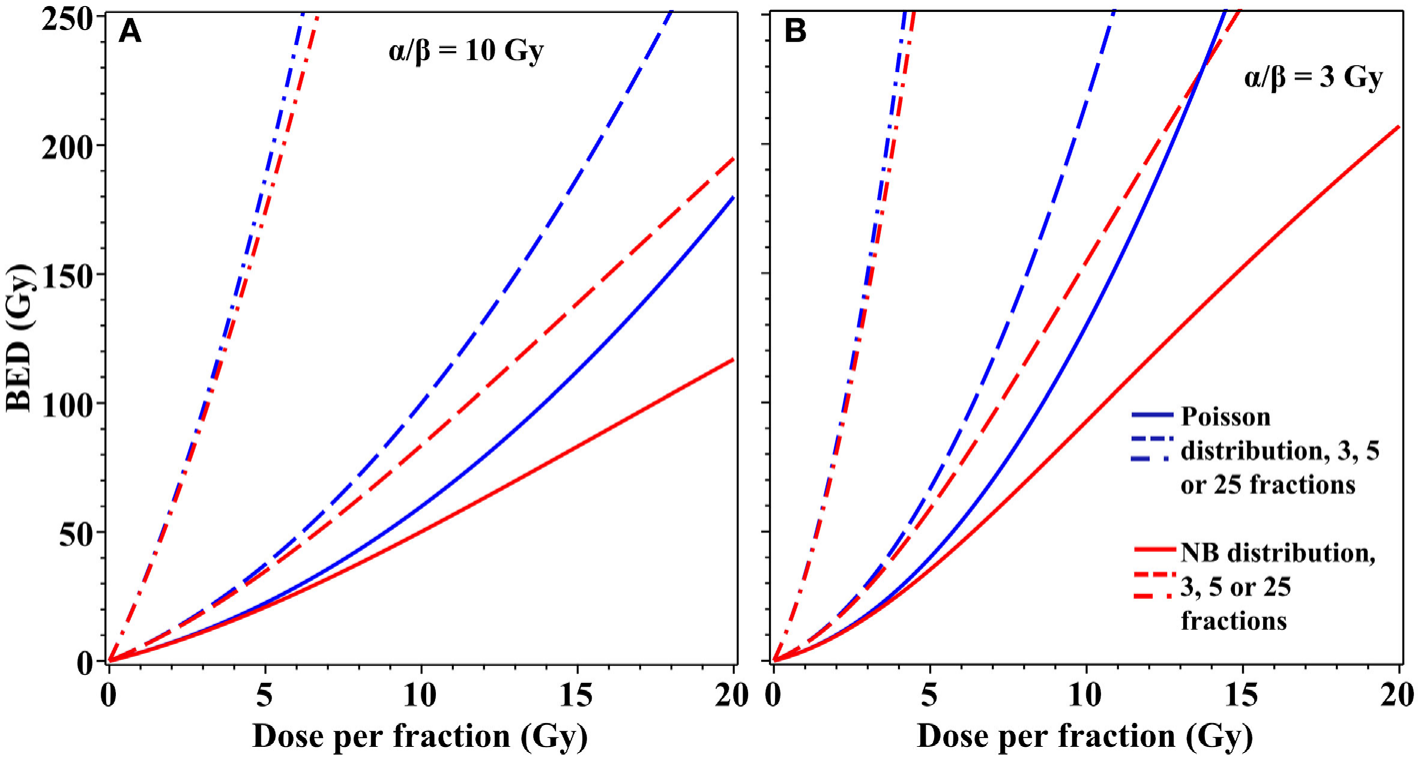
Effects of overdispersion of lethal lesions on biologically effective dose (BED) estimates for fractionated radiotherapy. Blue curves, Poisson distribution; red curves, NB distribution with the overdispersion parameter *r* = 0.138, the best-fit value for data on lethal lesions in lymphocytes exposed to 4 Gy of gamma rays. Details of the BED calculations are described in the main text. **(A)** α/β = 10 Gy; **(B)** α/β = 3 Gy. Solid curves, 3 dose fractions; dashed curves, 5 fractions; dash-dotted curves, 25 fractions.

The differences in cell survival predicted by using an overdispersed distribution instead of the Poisson distribution can be important not only for single-dose radiotherapy but also for fractionated stereotactic treatments. For example, moderate overdispersion of lethal lesions per cell modeled by the NB distribution substantially modifies BED estimates for radiotherapy regimens that use three to five dose fractions of ≥5 Gy/fraction (Figure 4). Taking these effects into consideration may be useful for optimization of radiotherapy treatment planning to maximize tumor control while minimizing normal tissue toxicity.

Our results do not contradict other explanations for cell survival curve “straightening” at high doses because an overdispersed error distribution and non-LQ dose dependence for the mean number of lethal lesions per cell are not mutually exclusive. Therefore, we do not argue specifically in favor of the LQ formalism and/or of the NB distribution. Our point is that even a continuously curving dose–response formalism coupled with an overdispersed error distribution is capable of accounting for survival curve “straightening” at high doses without invoking complicated or *ad hoc* explanations for the dose dependence of the mean lethal lesion yield. Taking the error distribution into consideration may be a tractable and clinically useful approach for modeling the effects of modern radiotherapy with high doses per fraction.

## Author Contributions

All authors: study design, data analysis, and manuscript writing.

## Conflict of Interest Statement

The authors declare that the research was conducted in the absence of any commercial or financial relationships that could be construed as a potential conflict of interest. The reviewers TJ and DP and the handling editor declared their shared affiliation.

## Appendix

The customized negative binomial (NB) distribution, where *P*_NB_(*k*) is the probability of observing *k* lethal lesions in a cell, Γ is the gamma function, and *r* is the “overdispersion” parameter, is described as follows:

(A1) $$P_{\text{NB}}(k)={\left(1/[r\times Q]\right)}^{1/r}\times {\left(Y/Q\right)}^{k}\times \Gamma (k+1/r)/[\Gamma (1/r)\times k!],Q=Y+1/r.$$

The Poisson log-likelihood can be written as:

(A2) $${\text{LL}}_{\text{Pois}}=k\times \text{ln}[Y]-Y-\text{ln}[k!].$$

The NB log-likelihood can be written as:

(A3) $$\begin{array}{ll} {\text{LL}}_{\text{NB}}= & -\left(\frac{1}{r}\right)\times (1+k\times r)\times \text{ln}[1+r\times Y]+k\times (\text{ln}[r]+\text{ln}[Y])\\ & +\text{ln}\left[\Gamma \left(\frac{1+k\times r}{r}\right)\right]-\text{ln}[\Gamma (1+k)]-\text{ln}\left[\Gamma \left(\frac{1}{r}\right)\right]. \end{array}$$

Binomial approximations for the Poisson (BLL_Pois_) and NB (BLL_NB_) distributions, which were used to analyze fibroblast data, are described as follows:

(A4) $$\begin{array}{ll} {\text{BLL}}_{\text{Pois}} & =N\times P0_{\text{obs}}\times \text{ln}[\text{exp}[-Y_{\text{obs}}]]+N\times (1-P0_{\text{obs}})\times \text{ln}[1-\text{exp}[-Y_{\text{obs}}]],\\ {\text{BLL}}_{\text{NB}} & =N\times P0_{\text{obs}}\times \text{ln}\left[{\left(1+r\times Y_{\text{obs}}\right)}^{\left(-\frac{1}{r}\right)}\right]+N\times (1-P0_{\text{obs}})\times \text{ln}\left[1-{\left(1+r\times Y_{\text{obs}}\right)}^{\left(-\frac{1}{r}\right)}\right]. \end{array}$$

Here, BLL represents the Binomial log-likelihood, *N* is the number of analyzed cells at the given radiation dose, *P0*_obs_ is the observed fraction of cells with zero lethal lesions, and *Y*_obs_ is the observed mean number of lethal lesions per cell.

Akaike information criterion with sample size correction (AICc) for the *M*th-modeled distribution (AICc*_M_*) is given below, where *Q_M_* is the number of adjustable parameters, LL*_M_* is the maximized log-likelihood value for the *M*th distribution (e.g., calculated using Eq. A2 or Eq. A3) for the entire data set, and *N*_tot_ is the total number of analyzed cells for all radiation doses in the data set:

(A5) $${\text{AICc}}_{M}=-2\times {\text{LL}}_{M}+2\times Q_{M}+2\times Q_{M}\times (Q_{M}+1)/(N_{\text{tot}}-Q_{M}-1).$$

The distribution with the lowest AICc value is considered to be best supported among those considered. The relative likelihood of the *M*th distribution, called the evidence ratio (ER*_M_*), can be expressed as follows:

(A6) $${\text{ER}}_{M}=\text{exp}\left[-\frac{{\text{ΔAICc}}_{M}}{2}\right],{\text{ΔAICc}}_{M}={\text{AICc}}_{M}-{\text{AICc}}_{\text{min}}\text{.}$$

Here, AICc_min_ is the lowest AICc value generated by the set of distributions being compared.

The evidence ratio for the tested distribution divided by the sum of the evidence ratios for all distributions being compared is the Akaike weight, *W_M_*. It represents the probability that the *M*th distribution would be considered best supported (among those tested) upon repeated sampling of the data. It is calculated as follows:

(A7) $$W_{M}={\text{ER}}_{M}/\sum_{M}{\text{ER}}_{M}.$$
